# An association with hypopituitarism and 9q subtelomere deletion syndrome

**DOI:** 10.1002/ccr3.1591

**Published:** 2018-10-25

**Authors:** Shinji Higuchi, Masaki Takagi, Ryojun Takeda, Hiroshi Yoshihashi, Satoshi Narumi, Tomonobu Hasegawa

**Affiliations:** ^1^ Department of Pediatric Endocrinology and Metabolism Children’s Medical Center Osaka City General Hospital Osaka Japan; ^2^ Department of Pediatrics Keio University School of Medicine Tokyo Japan; ^3^ Kojiya Child Clinic Tokyo Japan; ^4^ Department of Medical Genetics Tokyo Metropolitan Children’s Medical Center Tokyo Japan; ^5^ Department of Molecular Endocrinology National Research Institute for Child Health and Development Tokyo Japan

**Keywords:** 9q subtelomere deletion syndrome, array comparative genomic hybridization, central adrenal insufficiency, hypopituitarism, whole‐exome sequence

## Abstract

Hypopituitarism could have been overlooked so far in the patients with 9q subtelomere deletion syndrome (9qSTDS); thus, further investigations or reevaluation of clinical information, especially hormonal evaluations, are warranted to determine whether hypopituitarism is a rare or relatively common presentation in patients with 9qSTDS.

## 1 INTRODUCTION

The chromosome 9q subtelomere deletion syndrome (9qSTDS; MIM #610253), also known as Kleefstra syndrome, is one of the most common clinically recognizable subtelomere deletion syndromes, with approximately 100 reported cases in the literature.^1^, ^2^ The 9qSTDS is characterized by intellectual disability, severe hypotonia with speech and gross motor delay, and characteristic facial features, including micro‐ or brachycephaly, hypertelorism, synophrys, arched eyebrows, midface hypoplasia, a short nose with upturned nares, a protruding tongue, everted lower lip, and downturned corners of the mouth.^3^

In addition, congenital heart defects, epilepsy, and urogenital defects are frequently observed. To the best of our knowledge, however, hypopituitarism with 9qSTDS has not been previously reported. Here, we present the first case of definite hypopituitarism with a 9q34.3 subtelomeric deletion, thus expanding our understanding of the phenotypic features associated with 9qSTDS.

## 2 CASE REPORT

The patient was a 7‐year‐old Japanese boy born at 38 weeks of gestation after an uncomplicated pregnancy and delivery. His parents were nonconsanguineous and phenotypically normal. He had no family history of pituitary dysfunction. His birth length was 51.0 cm (1.0 SD), and weight was 3.4 kg (0.9 SD). At birth, several dysmorphic features including hypertelorism, synophrys, midface hypoplasia, right preauricular pits, prominent antihelix, short philtrum, and thin upper lip with downturned corners of the mouth were observed. Echography revealed an atrial septal defect and left renal hydronephrosis. An auditory brainstem response examination revealed mild hearing impairment in both ears. The testes were undescended. Neonatal screening levels of thyroid‐stimulating hormone (TSH) and free T4 were normal. At the age of 1 years and 5 months, his height was 77.2 cm (−0.9 SD), weight was 8.35 kg (−1.8 SD), and head circumference was 44.6 cm (−1.3 SD), respectively.

**Figure 1:**
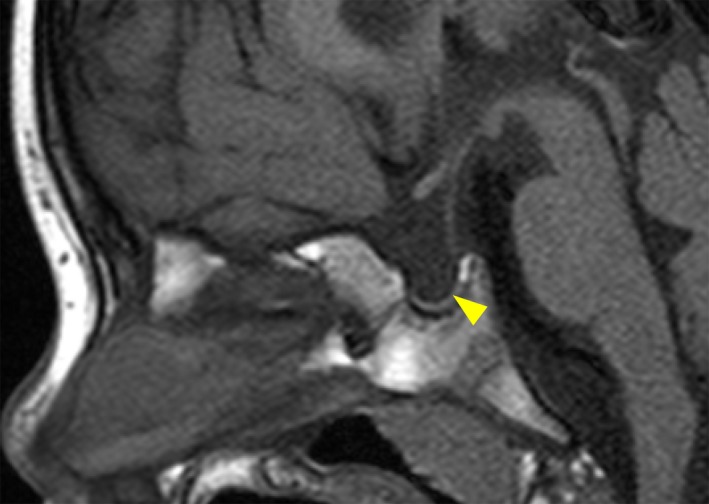
Brain magnetic resonance imaging of the patient at the age of 4 years. A hypoplastic anterior pituitary gland (arrowhead), with a visible but thin stalk, and an eutopic posterior pituitary gland are shown

He was referred to us at 4 years of age because of his short stature. His height and weight were 87.7 cm (−3.2 SD) and 10.2 kg (−2.7 SD), respectively. He showed micropenis, with a stretched penile length of 2.5 cm. Brain magnetic resonance imaging revealed an anterior pituitary hypoplasia with a visible but thin stalk, as well as an eutopic posterior pituitary gland (Figure 1). No other abnormalities in the central nervous system were found. Hormonal data revealed growth hormone (GH) and TSH deficiencies (Table 1). The cortisol peak response during the corticotropin‐releasing hormone (CRH) test was normal (peak cortisol = 17.3 μg/dL, Ref > 20.0 μg/dL or increment of 10 μg/dL). A replacement therapy with l‐thyroxine was started. Replacement therapy with GH was refused by the parents.

**Table 1:** Endocrinological findings in the patient

	Stimulus	4 years			6 years			Reference	
		Basal		Peak	Basal		Peak	Basal	Peak
GH (ng/mL)	Arginine	0.67	→	3.69	0.8	→	1.5		>6
TSH (mIU/mL)	TRH	2.127	→	31.117^a^					10‐35
PRL (ng/mL)	TRH	5.19	→	72.04				1.7‐15.4	Increase 2 times
ACTH (pg/mL)	CRH	16.5	→	40.1	14.8	→	51.4	9.8‐27.3	28‐130.5
Cortisol (μg/dL)	CRH	6.1	→	17.3	5.4	→	13.3	5‐20	>20.0 or increase >10
LH (mIU/mL)	GnRH	<0.1	→	3.42	<0.1	→	3.83	<0.1^b^	<0.10‐4.29^b^
FSH (mIU/mL)	GnRH	1.0	→	13.08	1.0	→	18.21	0.46‐1.43^b^	5.38‐11.67^b^
IGF‐1 (ng/mL)		13			16			32‐176^c^55‐215^d^	
Free T4 (ng/dL)		0.86						1.01‐1.95	
Free T3 (pg/mL)		3.14						2.23‐5.30	

ACTH, adrenocorticotropic hormone; CRH, corticotropin‐releasing hormone; FSH, follicle‐stimulating hormone; GH, growth hormone; GnRH, gonadotropin‐releasing hormone; IGF, insulin‐like growth factor; LH, luteinizing hormone; PRL, prolactin; TRH, thyrotropin‐releasing hormone; TSH, thyroid‐stimulating hormone; T3, triiodothyronine; T4, thyroxine.

The conversion factors to the SI unit are as follows: GH 1.0 (μg/L), TSH 1.0 (mIU/L), LH 1.0 (IU/L), FSH 1.0 (IU/L), testosterone, 0.035 (nmol/L), prolactin 1.0 (μg/L), ACTH 0.22 (pmol/L), cortisol 27.59 (nmol/L), IGF‐I 0.131 (nmol/L), free T4 12.87 (pmol/L), and free T3, 1.54 (pmol/L).

a The peak of TSH was normal; however, prolonged increase in TSH (20.241 mIU/mL at 120 minutes after TRH administration) indicated hypothalamic hypothyroidism.

b Reference data of UK children (younger than 10 y).

c Reference data of Japanese 4‐year‐old boys.

d Reference data of Japanese 6‐year‐old boys.

At the age of 6 years and 4 months, the patient exhibited hypoglycemia without an apparent cause. The blood glucose level was 35 mg/dL when he was admitted to our hospital. Hyperinsulinism was excluded based on the analysis of critical samples (serum insulin levels were below 0.60 μIU/mL). We reevaluated his pituitary function by provocation tests, and an impaired cortisol response was observed during the CRH test (peak cortisol 13.3 μg/dL). He was then diagnosed with central adrenal insufficiency due to adrenocorticotropic hormone (ACTH) deficiency (Table 1), and a replacement therapy with hydrocortisone was started. After starting hydrocortisone therapy, hypoglycemia has not been recorded since. At his last examination at the age of 7 years and 6 months, his height and weight were 106.0 cm (−3.2 SD) and 16.4 kg (−1.8 SD), respectively. Owing to severe psychomotor retardation, he remains wheelchair‐bound and nonverbal.

**Figure 2:**
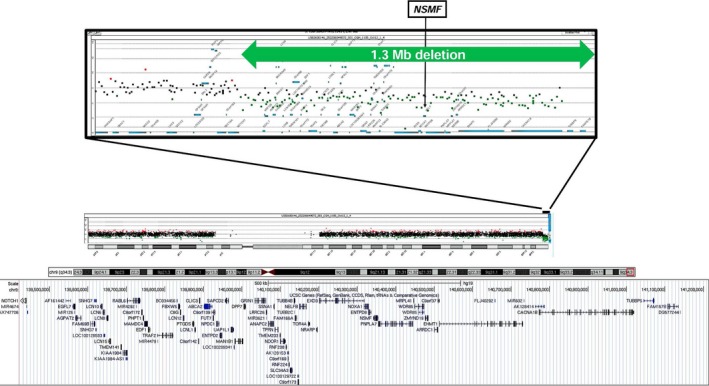
Result of array CGH analysis. Graphical representation of the results from the array CGH analysis (Agilent 4 × 180 K SurePrint G3 Human CGH Microarray) shows approximately 1.3‐Mb deletion of the 9q subtelomere. At the proximal breakpoint (centromeric), the last present probe was A_14_P113317 (chr 9:139432550‐139432609), while the first deleted probe was A_16_P02194382 (chr 9:139450604‐139450661). Base pair positions were derived from the University of California Santa Cruz (UCSC) Genome Browser (available on line at: http://genome.ucsc.edu/cgi-bin/hgGateway), build GRCh37 (hg19)

Genomic DNA from the patient was subjected to array comparative genomic hybridization with the Agilent 4 × 180 K SurePrint G3 Human CGH Microarray (catalog no. G4449A; Agilent Technologies). We identified a heterozygous 1.3‐Mb subtelomeric deletion at 9q34.3 (Figure 2). Multiplex ligation‐dependent probe amplification (MLPA) analysis of the parents revealed that this deletion was de novo (data not shown). The list of the deleted genes is provided as Data S1.

The combination of two relatively rare conditions, 9qSTDS and hypopituitarism, led us to perform additional study: whole‐exome sequence (WES) to obtain additional genetic information for the etiology of the hypopituitarism. Detailed are described in Data S3. We provide the list of variants of unknown significance (VUS) detected in WES as Data S2. The number of variants remaining after each filtering step is provided in Supplemental Table 1. No mutations were found in the currently known hypopituitarism‐related genes (*POU1F1*,* PROP1*,* HESX1*,* LHX4*,* OTX2*,* SOX2*,* SOX3*,* GLI2*,* PAX6*,* IGSF1*,* GPR161*,* FGF8*,* KAL1*,* PROK2R*, and *LHX3*). As one allele was lost for the region of chromosome 9q34.3 in the patient, any mutations in this region in the remained allele could be functionally null. However, we were unable to identify any pathological sequence variations in the genes located in the deleted region. Trio de novo approach using the DNAs from parents was refused.

## 3 DISCUSSION

To the best of our knowledge, this is the first example of a patient presenting with hypopituitarism, harboring a heterozygous 1.3‐Mb deletion in 9q34.3. Short stature and genital abnormalities, such as cryptorchidism and micropenis, both of which could be the results of hypopituitarism, are relatively common phenotypes in the patient with 9qSTDS: short stature in 32% and genital abnormalities in 32% of 9qSTDS patients.^4^ No 9qSTDS cases evaluated for pituitary functions have been reported; therefore, hypopituitarism with 9qSTDS could have been overlooked so far, and further investigations or reevaluations of clinical information, especially hormonal evaluations, are warranted. It is also of note that the secretion of ACTH gradually decreased in our patient. A gradual loss of ACTH is a point of concern in some patients with congenital hypopituitarism. Therefore, we suggest careful follow‐up monitoring of the hypothalamic‐pituitary‐adrenal function in patients with 9qSTDS and hypopituitarism as adrenal insufficiency could be lethal, even if ACTH deficiency is not apparent during the first evaluation.

The combination of two relatively rare conditions, 9qSTDS and hypopituitarism, led us to perform WES to obtain additional genetic information for hypopituitarism; however, we were unable to identify any mutations in the known causative genes for hypopituitarism or second hit mutations in genes located in the deleted region. Hypogonadotropic hypogonadism (HH), which results in micropenis and cryptorchidism, in patients with 9qSTDS could be due to a haploinsufficiency of *NSMF* (MIM # 608137). This gene is located at chr9:140342023‐140353786 in the commonly deleted region, encodes a guidance molecule for olfactory axon projections, and plays a role in the neurophilic migration of luteinizing hormone‐releasing hormone cells.^5^ Several reports have shown that *NSMF* is associated with normosmic idiopathic HH and Kallmann syndrome (KS), defined by HH and anosmia/hyposmia, either singly or in combination with a mutation in another gene.^6^, ^7^, ^8^, ^9^ Recent studies have shown that variants in *FGF8*,* KAL1,* and *PROKR2*, the genes responsible for KS, have been identified in a small number of hypopituitarism.^10^, ^11^ Haploinsufficiency of *NSMF* could partly contribute to pituitary development. Of course, the possibility that hypopituitarism and 9qSTDS are coincident, and that the patient had additional variants in unidentified genes causative for hypopituitarism, cannot be completely excluded.

In conclusion, we present the first case of definite hypopituitarism with a 9q34.3 subtelomeric deletion, thus expanding our understanding of the phenotypic features associated with 9qSTDS. However, hypopituitarism could have been overlooked so far in the patients with 9qSTDS; thus, further investigations or reevaluation of clinical information, especially hormonal evaluations, are warranted to determine whether hypopituitarism is a rare or relatively common presentation in patients with 9qSTDS.

## CONFLICT OF INTEREST

The authors have nothing to declare.

## AUTHOR CONTRIBUTION

Shinji Higuchi, Masaki Takagi, and Tomonobu Hasegawa: conceived and designed the experiments. Masaki Takagi and Satoshi Narumi: performed the experiments and analyzed the data. Shinji Higuchi, Ryojun Takeda, and Hiroshi Yoshihashi: contributed reagents/materials/analysis tools. Shinji Higuchi and Masaki Takagi: wrote the manuscript.
