# Supplementary material for: An association with hypopituitarism and 9q subtelomere deletion syndrome
Source: Clin Case Rep. 2018 Oct 25;6(12):2371–5. doi: 10.1002/ccr3.1591 (PMC6293262; doi:10.1002/ccr3.1591)
Supplement: Supplementary file 3 [file CCR3-6-2371-s003.doc]

**Data S3. Supplemental Methods**

**Whole exome sequence (WES)**

With informed consent and with approval of the Institutional Review Board of Keio University School of Medicine, genomic DNA was extracted from peripheral blood leucocytes of the patient and his parents.

DNA was captured with a SureSelect Human All Exon V5 Kit (Agilent Technologies) and sequenced on a HiSeq 2500 sequencer (Illumina, San Diego, CA) with 100-bp paired end reads. Image analysis and base calling were performed by sequence control software real-time analysis (RTA) v1.18.61, and CASAVA software v1.8.2 (Illumina). We used BWA 0.7.10 and SAM tools 0.1.18 for alignment and variant detection against the human reference genome (NCBI build 37; hg19). Variant calling as well as coverage and depth calculations were performed with the Genome Analysis Toolkit 3.3-0 (http://www.broadinstitute.org/gatk/). The resulting single-nucleotide variants were annotated with ANNOVAR (Center for Applied Genomics, Children's Hospital of Philadelphia, Philadelphia, PA).

Only nonsense, non-synonymous, splice site variants, synonymous splice site variants, insertion, and deletion variants that affect coding regions of gene were used for interpretation. Common variants registered in the 1000 Genomes Project (with minor allele frequency of >0.01), in ExAC databases (with minor allele frequency of >0.01), and the Human Genetic Variation Database (http://www.genome.med.kyoto-u.ac.jp/SnpDB/) in Japan, were removed.
