# Supplementary material for: An association with hypopituitarism and 9q subtelomere deletion syndrome
Source: Clin Case Rep. 2018 Oct 25;6(12):2371–5. doi: 10.1002/ccr3.1591 (PMC6293262; doi:10.1002/ccr3.1591)
Supplement: Supplementary file 4 [file CCR3-6-2371-s004.docx]

Table S1. The result of whole exome sequence (filtering).

| Total variants | 78,997 |
| --- | --- |
| Registered variants in the 1000 Genomes Project (with minor allele frequency of >0.01), in ExAC databases (with minor allele frequency of >0.01) , and Human Genetic Variation Database (http://www.genome.med.kyoto-u.ac.jp/SnpDB/) in Japan were removed. | 2810 |
| Within coding genes | 231 |
| Only nonsense, non-synonymous, splice site variants | 129 |
| Heterozygous | 126 |
| Homozygous | 3 |
